# Views on help-seeking for psychosis in South and Southeast Asia: A systematic mixed studies review

**DOI:** 10.1192/j.eurpsy.2025.2200

**Published:** 2025-08-26

**Authors:** G. E. Aurizki, R. Griffiths, L. Renwick

**Affiliations:** 1 Division of Nursing, Midwifery and Social Work, The University of Manchester, Manchester, United Kingdom; 2 Faculty of Nursing, Universitas Airlangga, Surabaya, Indonesia

## Abstract

**Introduction:**

Psychosis is a condition characterised by hallucinations or delusions, which can cause distress and burden for both the individuals experiencing it and their caregivers. Seeking help can reduce the distress and burden, improve symptoms, and prevent adverse outcomes. However, many people still do not seek help or delay help-seeking. The reasons for this are not yet fully understood, especially in South and Southeast Asian settings.

**Objectives:**

To explore the views and experiences of key stakeholders in South and Southeast Asia towards help-seeking for psychosis.

**Methods:**

This systematic review included ‘views’ studies using qualitative, quantitative, and mixed-method designs. Systematic searches were conducted in several databases, including CINAHL, MEDLINE, PsycINFO, Embase, ASSIA, and Scopus from inception to September 2023. Each article was screened in duplicate by at least two independent reviewers. Popay et al.’s (2006) narrative synthesis was used to synthesise the data.

**Results:**

This review included 51 studies from 10 South and Southeast Asian countries. The synthesis revealed that people with psychosis and their caregivers sought help from various sources. Factors that facilitate or hinder help-seeking behaviours include the ability to recognise problems, the alignment between illness and treatment perceptions, the attainability of help providers, the roles of social networks and support systems, and perceived stigma. Further, people also considered the outcome of each help-seeking process to determine their subsequent behaviours.

**Conclusions:**

Interventions to improve help-seeking behaviours among people with psychosis should consider these multiple factors and address not only the help-seekers but also a wider range of key stakeholders, including social networks and potential help providers.

**Disclosure of Interest:**

None Declared

